# Influence of Complex Disease on the Accuracy of Transvaginal Ultrasound Diagnosis of Uterosacral Ligament Endometriosis

**DOI:** 10.1002/ajum.70021

**Published:** 2025-09-28

**Authors:** Shay M. Freger, Ido Mick, Saar Aharoni, Mathew Leonardi

**Affiliations:** ^1^ Department of Obstetrics & Gynecology McMaster University Hamilton Ontario Canada; ^2^ Robinson Research Institute University of Adelaide Adelaide Australia

**Keywords:** diagnostics test accuracy, endometriosis, pouch of Douglas obliteration, transvaginal ultrasound, uterosacral ligaments

## Abstract

**Objective:**

To assess the impact of complex disease states, including pouch of Douglas (POD) obliteration and deep endometriosis (DE) of the bowel, on the diagnostic accuracy of transvaginal ultrasound (TVUS) for detecting endometriosis of the uterosacral ligaments (USLs) and torus uterinus (TU).

**Methods:**

This diagnostic accuracy study evaluated the performance of TVUS in diagnosing DE of the USLs and TU, using laparoscopic visualisation with histological confirmation as the reference standard among two previously reported prospectively collected cohorts. Complex disease states were defined as complete POD obliteration and/or DE of the bowel. Diagnostic accuracy metrics, including sensitivity, specificity, positive and negative predictive values (PPV) and likelihood ratios (LR), were calculated before and after the exclusion of complex disease cases.

**Results:**

Among 177 participants, 18.6% (33/177) had POD obliteration, 18.6% (33/177) had DE of the bowel and 16.4% (29/177) had both. Accuracy ranged from 93.1% to 94.7% for USLs and 97.2%–98.5% for TU, with minimal change after exclusion (≤ 1.5%). Sensitivity declined following exclusion, by −6.4% (left USL), −3.5% (right USL) and −2.7% (TU) after POD obliteration exclusion and further decreases of −1.8%, −3.4% and −4.4%, respectively, after bowel DE exclusion. Specificity remained ≥ 97.8% across all sites and reached 100% at the USLs after POD obliteration exclusion.

**Conclusions:**

Contrary to the assumption that complex disease states hinder TVUS accuracy, their presence may enhance lesion recognition, likely due to increased sonographic attentiveness when severe disease is suspected. While TVUS remains highly specific, its sensitivity decreases in the absence of complex disease, emphasising the need for meticulous and systematic imaging approaches.

**Summary**

- Endometriosis is a debilitating disease that affects millions of individuals worldwide, yet its diagnosis remains a significant challenge, particularly for deep endometriosis (DE) in the uterosacral ligaments (USLs) and torus uterinus (TU).
- Transvaginal ultrasound (TVUS) has emerged as a critical tool for preoperative mapping, but its accuracy in complex cases—where the pelvic anatomy is significantly distorted—has not been well understood.
- Our study challenges the assumption that severe disease hinders ultrasound performance.
- Instead, we demonstrate that complex disease states, such as pouch of Douglas obliteration and bowel DE, may enhance lesion recognition, likely due to heightened sonographic scrutiny in these cases.
- These findings underscore the need for meticulous imaging techniques and reinforce TVUS as an indispensable tool in the preoperative assessment of endometriosis, guiding surgical planning and improving patient outcomes.

## 1 Introduction

**FIGURE 1:**
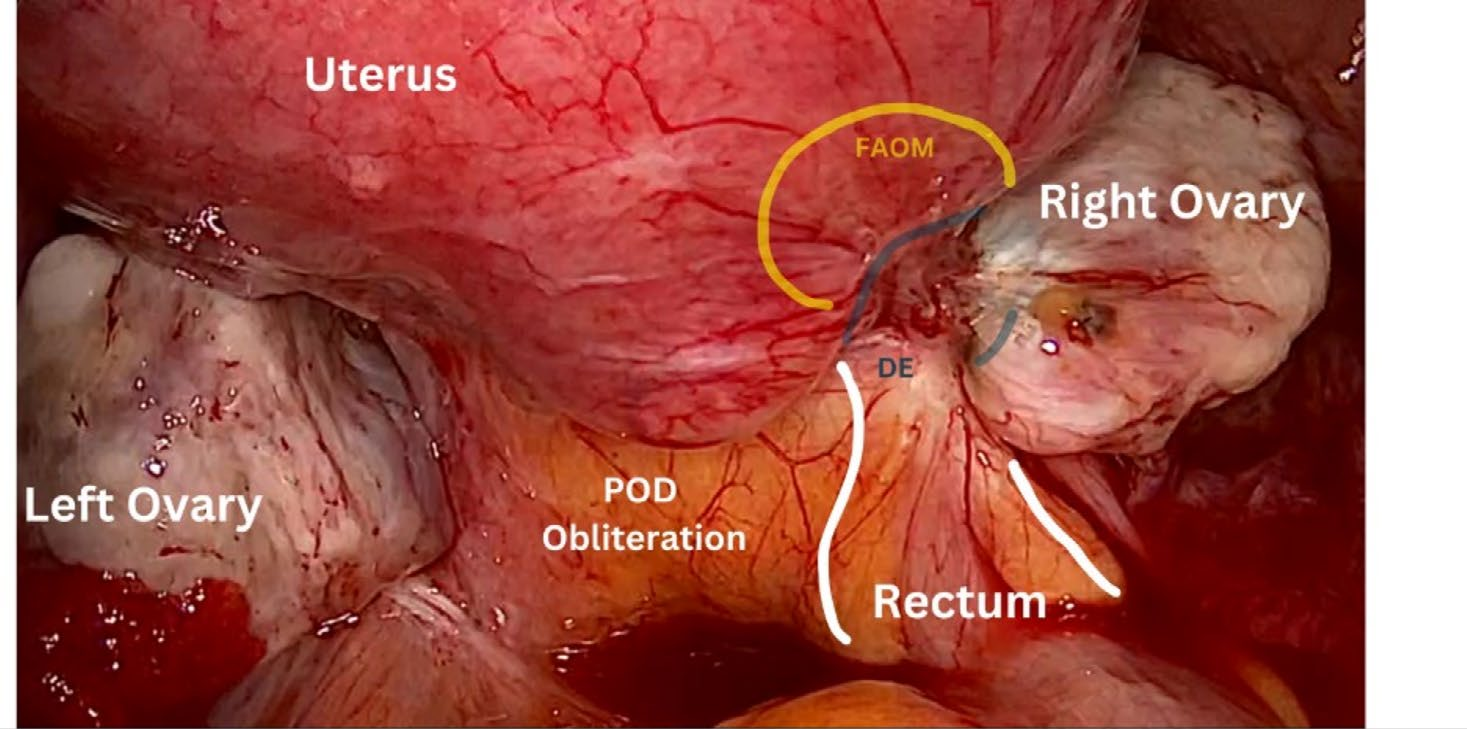
Laparoscopic view of a distorted pelvic environment due to the presence of DE and POD obliteration. DE, deep endometriosis; FOAM, focal adenomyosis of the outer myometrium; POD, pouch of Douglas.

Endometriosis is a chronic pain and inflammatory disease characterised by abnormal growth of endometrial‐like cells outside of the uterus, leading to significant negative impacts on quality of life [1]. Deep endometriosis (DE) is the most aggressive subtype, composing approximately 20% of all endometriosis diagnoses [2]. DE is characterised by its infiltrative nature within surrounding structures, leading to significant distortion of the surrounding anatomical milieu through adhesions and displacement of intrapelvic structures [3], depicted in Figure 1. The most common location for DE remains the uterosacral ligaments (USLs), bilateral connective structures between the uterus and sacrum, conjoined by the torus uterinus (TU) [4], creating the lateral boundaries of the pouch of Douglas (POD), the space between the bowel and uterus [5]. USLs may be affected by discrete DE nodules or contiguously, whereby bilateral structures may be affected with TU involvement [6].

In moderate and severe cases, such as those with stage III or IV endometriosis based on the revised American Society for Reproductive Medicine (rASRM) classification, USL nodules may co‐occur with highly prevalent pelvic adhesions [7], or be involved with additional disease locales, most commonly, the bowel. Alongside aggressive forms of endometriosis, the POD may be ‘obliterated,’ whereby dense adhesions are present between the uterus/retrocervix and rectum, leading to the absence of mobility between the posterior aspect of the uterus and bowel and an inability to surgically visualise the space between and inferior to the USLs [8, 9]. Without accurate pre‐operative mapping of DE lesions in these anatomically distorted states, there is a possibility that surgeons may be performing incomplete excision, unbeknownst to them or their patients. Incomplete excision of USL endometriosis may lead to the persistence or reoccurrence of symptoms such as deep dyspareunia, chronic pelvic pain and dyschezia. Moreover, residual disease often necessitates repeat surgeries, which carry cumulative surgical risks, increased healthcare costs and emotional and physical burden for the patient. Accurate identification of USL disease is therefore critical not only for intraoperative guidance but also for long‐term symptom relief and surgical efficiency.

Although the USLs remain invaluable structures and the most common locale for DE, they have been historically associated with low diagnostic accuracy sonographically. With improved accuracy noted upon developing the pivotal International Deep Endometriosis Analysis group (IDEA) consensus, allowing for standardised reporting of sonographic findings, the USLs and TU remained elusive [10]. Quite possibly, it may be that the severe anatomical distortion of the posterior compartment and complexity of sonographic evaluation in these cases negatively influence the diagnostic test accuracy, leading to missed disease and reduced sensitivity.

While several studies have elucidated the diagnostic accuracy of DE when multiple sites are impacted [4, 11, 12, 13], including the USLs, the impact of complex disease states and anatomical distortion on diagnostic accuracy remains unexplored. As such, we hypothesised that the presence of complex disease states reduces the diagnostic performance of TVUS in diagnosing DE of USLs and TU, leading to historically poor sensitivity. The objective of this study is to evaluate the impact of these complex disease states on the diagnostic accuracy of DE of the USLs and TU.

## 2 Methods

### 2.1 Study Design

The study is a prospective diagnostic test accuracy study to investigate the influence of complex disease states, including POD obliteration and DE of the bowel, on the diagnostic accuracy of TVUS in diagnosing DE of the USLs and TU. This study was done and reported per Standards for Reporting Diagnostic Accuracy (STARD; 2015) [14] guidelines for standardisation in diagnostic accuracy methodology and reporting, with consideration for the Quality Assessment of the Diagnostic Accuracy of Studies (QUADAS‐2) checklist [15]. The study was conducted at McMaster University Medical Centre, Hamilton Health Sciences. Data were prospectively collected in real time between August 2020 and January 2023.

### 2.2 Participants

Participants were prospectively recruited between August 2020 and January 2023 from the gynaecology and minimally invasive surgery clinics at McMaster University Medical Center, Hamilton Health Sciences. Consecutive sampling was used; all patients referred for surgical evaluation of suspected endometriosis who met eligibility criteria were invited to participate. The participant cohort involved those previously reported in two separate, non‐overlapping studies assessing the diagnostic test accuracy of TVUS in diagnosing DE of the USLs [6] and among all sites of endometriosis [11]. Participant data were collected consecutively, where the reference standard was performed within 1 year of the index test. Participant inclusion criteria included (1) age between 18 and 50 years, (2) assigned female sex at birth, (3) pre‐menopausal and post‐menarchal, (4) history of chronic pelvic pain and/or endometriosis, (5) able to undergo TVUS and consented to laparoscopic surgery for endometriosis. Participants were excluded if they had a current or previous gynaecologic malignancy, or if they underwent laparoscopy at a different centre. Participants without characterisable USLs and TU on TVS and surgically were excluded from analysis. In this study, ‘complex disease’ was defined as a distinct anatomical condition rather than a formal staging classification. It was operationalised based on the presence of either (1) complete obliteration of the pouch of Douglas (POD) or (2) deep endometriosis (DE) of the bowel, or both. These findings were determined intraoperatively, as they represent disease states that substantially distort pelvic anatomy and may impact sonographic detection.

### 2.3 Ethics Statement

The study was approved by the Hamilton integrated Research Ethics Board (HiREB: 12617).

### 2.4 Index Test and Reference Standard

The index test was TVUS using a standardised posterior approach and reported per the IDEA consensus [9], and the reference standard was laparoscopic visualisation and histology when tissue was available, which was performed by a single expert surgeon‐sonologist. The surgeon‐sonologist performing the index and reference tests was considered an expert based on the European Federation of Societies for Ultrasound in Medicine and Biology (EFSUMB) (level 3) and is certified in Minimally Invasive Gynaecologic Surgery (MIGS) [16]. The index test was performed in parallel to those previously described [6, 11], with the probe positioned in the posterior vaginal fornix, and the operator sequentially rotated the probe ≤ 45° clockwise to assess the left USL and counterclockwise for the right USL, tracing the hyperechoic peritoneum laterally to the expected ligament insertion sites. Hypoechoic nodules were identified by their disruption of the normal peritoneal boundary and by associated contour abnormalities or thickening [17, 18]. The TU was assessed at midline, immediately posterior to the cervix, and was visualised as a thin, linear hyperechoic band bridging the bilateral USLs. All structures were evaluated with dynamic transducer pressure to navigate anatomical distortion. Cine‐loop recordings and still images were captured during dynamic sweeping movements to maximise lesion conspicuity. All ultrasound assessments were performed following the four‐step pelvic scanning sequence recommended by the IDEA consensus. This included evaluation of the uterus and adnexa, identification of soft markers (such as site‐specific tenderness and ovarian mobility), assessment of POD obliteration status and evaluation of the anterior and posterior compartments. All TVUS scans adhered to the IDEA consensus standards for diagnoses and documentation [9]. Surgically, DE was characterised by a nodule portraying diverse pigmentation and a firm tactile response upon palpation [2].

### 2.5 Identification of Complex Disease States

Both complex disease states were assessed sonographically and confirmed surgically with or without histological confirmation if tissue was made available for DE of the bowel. POD obliteration was assessed on TVUS using the ‘sliding sig’ method in assessing mobility between the posterior aspect of the uterus and the bowel [9]. Obliteration was confirmed surgically by the inability to visualise the peritoneum of the POD, between and inferior to the USLs, due to adhesions between the uterus and bowel [9]. Partial obliteration, defined as a partial visualisation of the peritoneum of the POD, was considered *non‐complex disease* and grouped within the non‐complex cohort. Obliteration was treated through adhesiolysis, and DE of the bowel was excised for histological confirmation among those who consented to complete excision of disease.

The presence of DE of the bowel was characterised with any infiltration of a nodule into the muscularis of the bowel, per updated terminology for DE [2]. To be included in this *complex disease state*, patients needed surgically visualised/palpated bowel DE (including histological confirmed DE of the bowel when tissue was available following colorectal surgical excision). Bowel DE was appreciable at surgery in the location of most dense adhesions and palpable with the bowel graspers at the site of fibrotic (i.e., hard) nodularity within the anterior bowel wall [9].

### 2.6 Sample Size

This study utilised data from two previously published datasets evaluating the diagnostic test accuracy of TVUS for detecting DE among anatomical sites [6, 11]. After applying exclusion criteria, a total of 177 participants were included. A post hoc power analysis was conducted using IBM SPSS Statistics version 29 (SPSS Inc., Chicago, IL, USA) to determine the minimum effect size that could be detected with adequate power. The baseline sensitivity of TVUS for USL‐DE in this cohort ranged from 80.1% to 94.4% across anatomical sites. With 177 participants, the study had 80% power to detect a 13.0% absolute difference in sensitivity at an alpha level of 0.05. Given the study's descriptive aim and sample size, no formal statistical testing was conducted to compare diagnostic parameters across subgroups. Instead, changes in diagnostic accuracy metrics are described using absolute differences and overlapping 95% confidence intervals, in alignment with STARD 2015 guidance.

### 2.7 Analysis

The prevalence of POD obliteration and DE of the bowel were collected and reported. Baseline diagnostic accuracy parameters for bilateral USLs and TU, including accuracy, sensitivity, specificity, negative predictive value (NPV) and positive predictive value (PPV), negative likelihood ratio (LR−) and positive likelihood ratio (LR+) with 95% confidence interval (CI), were calculated. To assess the impact of *complex disease states* on the diagnostic test accuracy of TVUS in diagnosing DE of the USLs and TU, parameters were calculated: (1) excluding those with complete POD obliteration and (2) excluding those with DE of the bowel. Both complex disease states were descriptively compared individually to the reported baseline parameters and CIs. Participants with missing or indeterminate data, specifically those with uncharacterisable USLs or TU on either TVUS or at surgery, were excluded from the analysis. No imputation was performed.

Data were collected using the REDCap electronic data capture tool (Vanderbilt University, Nashville, Tennessee, USA) and imported into Microsoft Excel for Windows 10 (Microsoft Corporation, Santa Rosa, CA, USA). Cleaned data were transferred and analysed using IBM SPSS statistics V29 software (SPSS Inc., Chicago, IL, USA). All accuracy parameters were determined using the cross‐tabulation function in SPSS.

## 3 Results

### 3.1 Surgical and Histological Findings

**FIGURE 2:**
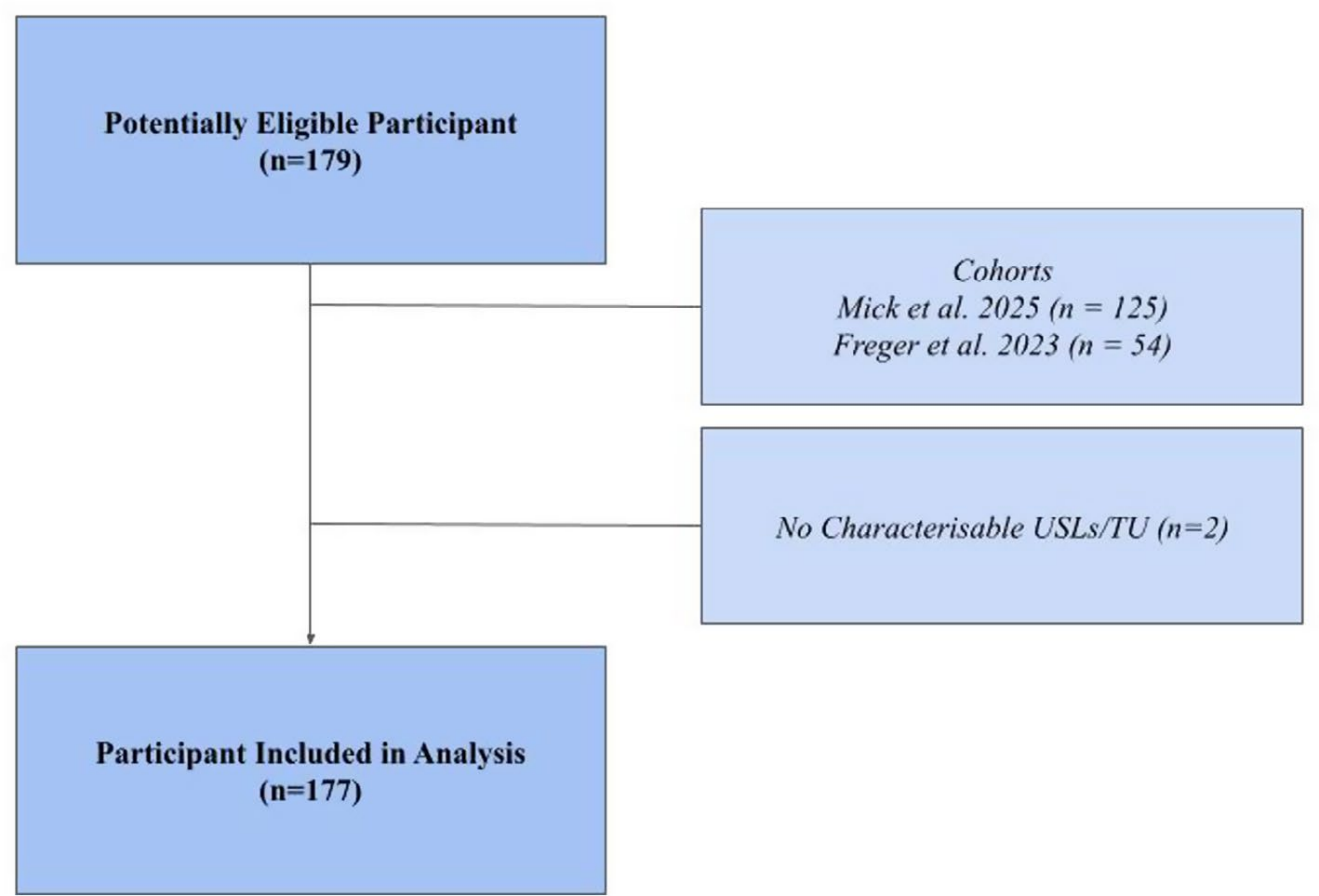
Study flowchart depicting participant inclusion and exclusion. *N*, number, TU, torus uterinus; USL, uterosacral ligament.

Among 179 eligible participants, two were excluded due to non‐characterisable USLs/TU, and 177 were included in the final analysis (Figure 2). Participant characteristics included in the final analysis are reported in Table 1.

**TABLE 1:** Characteristics of included participants (*N* = 177).

Characteristic	Value
**Age (Mean (SD))**	36.2 (±) 6.9
**Gravidity (% (*n*/*N*))**	
0	47.5 (84/177)
1	18.1 (32/177)
2	15.2 (27/177)
3+	19.2 (34/177)
**Parity (% (*n*/*N*))**	
0	62.1 (110/177)
1	13.0 (23/177)
2	15.8 (28/177)
3+	9.1 (16/177)
**Previous surgery (% (*n*/*N*))**	
Cesarean	15.3 (27/177)
Endometriosis	33.9 (60/177)
**Symptoms (% (*n*/*N*))**	
Dysmenorrhea	89.3 (158/177)
Chronic pain	73.4 (130/177)
Dyspareunia	62.1 (110/177)
Dyschezia	55.9 (99/177)
Abnormal uterine bleeding	53.1 (94/177)
Infertility	30.5 (54/177)
Dysuria	24.3 (43/177)

Prevalence of complete POD obliteration was 18.6% (33/177), which was assessed sonographically and confirmed laparoscopically. Among participants, 12.4% (22/177) had partial obliteration of the POD and were included in the *non‐complex cohort*. Laparoscopically confirmed DE of the bowel was present in 18.6% (33/177) of participants, with 16.9% (30/177) being histologically confirmed and three specimens were not histologically confirmed as colorectal surgery was not performed. Though the complex disease states were evaluated individually, 16.4% (29/177) had both POD obliteration and DE of the bowel.

### 3.2 Diagnostic Performance

**FIGURE 3:**
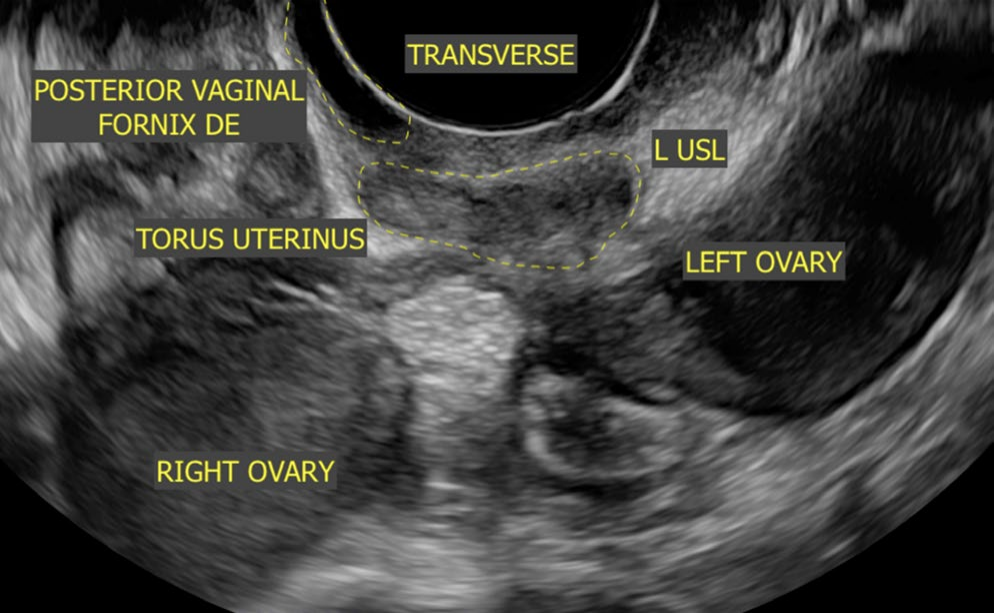
Sonographic characterisation of DE of the USLs and TU in the presence of POD obliteration in transverse. DE, deep endometriosis, LUSL, left uterosacral ligament; TU, torus uterinus.

The diagnostic accuracy of TVUS for the USLs and TU, stratified by the presence or absence of complex disease states, is summarised in Table 2. Sonographic characterisation of DE of the USLs and TU in the presence of complex disease states is as shown in Figure 3 and Video S1. Accuracy remained stable across all sites, with minimal changes following exclusion. For the left USL, accuracy was 93.2% (95% CI: 88.5–96.4) in the whole cohort, 93.2% (95% CI: 87.5–96.9) after POD obliteration exclusion (±0.0%) and 93.1% (95% CI: 87.6–96.6) after bowel DE exclusion (−0.1%). The right USL slightly increased after POD obliteration exclusion (94.7%, +1.5%) and remained stable after bowel DE exclusion (93.8%, +0.6%). The TU maintained the highest accuracy across groups, at 97.7% (95% CI: 94.3–99.4) in the whole cohort, 98.5% (95% CI: 94.6–99.8) after POD obliteration exclusion (+0.8%) and 97.2% (95% CI: 93.0–99.2) after bowel DE exclusion (−0.5%).

**TABLE 2:** Impact of surgically confirmed POD obliteration and DE of the bowel on the diagnostic accuracy of TVUS diagnosis of DE of bilateral USLs and TU relative to baseline performance.

	Left USL			Right USL			TU		
	Whole (*n* = 54%; 95% CI)	(X) POD (*n* = 41%; 95% CI)	(X) Bowel (*n* = 44%; 95% CI)	Whole (*n* = 54%; 95% CI)	(X) POD (*n* = 41%; 95% CI)	(X) Bowel (*n* = 44%; 95% CI)	Whole (*n* = 54%; 95% CI)	(X) POD (*n* = 41%; 95% CI)	(X) Bowel (*n* = 44%; 95% CI)
Accuracy	93.2% (88.5–96.4)	93.2% (87.5–96.9)	93.1% (87.6–96.6)	93.2% (88.5–96.5)	94.7% (89.4–97.8)	93.8% (88.5–97.1)	97.7 (94.3–99.4)	98.5% (94.6–99.8)	97.2% (93.0–99.2)
Sensitivity	87.3% (77.9–93.8)	80.9% (66.7–90.9)	85.5% (73.3–93.5)	80.9% (69.1–89.8)	77.4% (58.9–90.4)	77.5% (61.6–89.2)	94.4% (81.3–99.3)	91.7% (61.5–99.8)	90.0% (68.3–98.8)
Specificity	97.9% (92.8–99.7)	100% (95.8–100)	97.8% (92.1–99.7)	100% (96.8–100)	100% (96.4–100)	100% (96.5–100)	98.6% (94.9–99.8)	99.2% (95.4–99.9)	98.4% (94.3–99.8)
PPV	97.2% (89.7–99.3)	100% (90.8–100)	95.9% (85.6–98.9)	100% (93.0–100)	100% (85.6–100)	100% (88.8–100)	94.4 (81.1–98.5)	91.7% (60.8–98.7)	90.0% (69.3–97.3)
NPV	90.6% (84.3–94.5)	80.5 (84.2–94.5)	91.6% (85.1–95.4)	90.5 (85.1–94.1)	93.5% (88.3–96.5)	92.0% (86.7–95.4)	98.6% (94.8–99.6)	99.2% (94.8–99.9)	98.4% (94.2–99.6)
LR+	42.8 (10.8–169.2)	—	38.0 (9.6–150.3)	—	—	—	66.6 (16.8–264.2)	110.0 (15.5–780.3)	55.4 (13.9–220.5)
LR−	0.1 (0.07–0.2)	0.2 (0.1–0.3)	0.1 (0.08–0.3)	0.2 (0.1 to 0.3)	0.2 (0.1 to 0.4)	0.2 (0.1–0.4)	0.06 (0.01–0.2)	0.08 (0.01–0.5)	0.1 (0.03–0.4)

Abbreviations: CI = confidence interval, LR− = negative likelihood ratio, LR+ = positive likelihood ratio, *n* = number, NPV− = negative predictive value, POD = pouch of Douglas, PPV− = positive predictive value, TU = torus uterinus, USL = uterosacral ligaments, X = excluded from analysis.

Sensitivity declined across all sites following exclusion. The left USL decreased from 87.3% (95% CI: 77.9–93.8) in the whole cohort to 80.9% (95% CI: 66.7–90.9) after POD obliteration exclusion (−6.4%) and 85.5% (95% CI: 73.3–93.5) after bowel DE exclusion (−1.8%). Similarly, the right USL dropped from 80.9% (95% CI: 69.1–89.8) to 77.4% (95% CI: 58.9–90.4) (−3.5%) and 77.5% (95% CI: 61.6–89.2) (−3.4%). The TU, with the highest baseline sensitivity, declined from 94.4% (95% CI: 81.3–99.3) to 91.7% (95% CI: 61.5–99.8) (−2.7%) and 90.0% (95% CI: 68.3–98.8) (−4.4%).

Specificity remained stable, reaching 100% for the USLs following POD obliteration exclusion, while the TU remained high at 98.6%–99.2%. PPV remained consistently high at 100% for the USLs after exclusion, while the TU PPV declined slightly from 94.4% (95% CI: 81.1–98.5) to 91.7% (95% CI: 60.8–98.7) (−2.7%) and 90.0% (95% CI: 69.3–97.3) (−4.4%). NPV exhibited the largest declines, particularly at the left USL (−10.1%, from 90.6% to 80.5%), whereas the right USL fluctuated slightly (+3.0% after POD obliteration exclusion, −1.5% after bowel DE exclusion). The TU remained largely stable (98.6%–98.4%).

## 4 Discussion

### 4.1 Main Findings

In this study, we assessed the influence of *complex disease states*, such as POD obliteration and DE of the bowel, on the accuracy of TVUS diagnoses of DE within the USLs and TU. Our findings demonstrate that excluding cases with complex disease states, including POD obliteration and DE of the bowel, had minimal impact on overall accuracy, specificity and PPV, while sensitivity and NPV were notably affected. Accuracy, specificity and PPV remained largely unchanged across all assessments. In contrast, sensitivity declined across all sites after exclusion, with the left USL decreasing from 87.3% by −6.4% (POD) and −1.8% (Bowel), the right USL from 80.9% by −3.5% and −3.4% and the TU from 94.4% by −2.7% and −4.4%, respectively. Similarly, NPV saw the greatest decline at the left USL (−10.1%), while changes at the right USL and TU were minimal. The findings indicate that TVUS, while maintaining high specificity, is less reliable for ruling out disease when complex disease states are absent.

### 4.2 Interpretation and Significance

The USLs remain the most common location of DE, with historically the lowest diagnostic accuracy among all anatomical sites described through TVUS [3, 4, 19]. Despite recent advancements, no studies have reported the influence of complex disease states on the diagnostic accuracy of TVUS for DE. Understanding the impact of these highly prevalent and co‐occurring disease states is pivotal in surgical planning and gauging disease severity sonographically. In the case of moderate and severe endometriosis, the anatomical environment is typically distorted due to adhesions [7, 20]. We hypothesised that this would lead to difficulties in mapping and characterising normal and diseased tissue, reducing diagnostic performance through missed disease, contributing to a lower sensitivity. Although we expected accuracy parameters to improve by removing diagnostically challenging cases, this did not occur. Instead, the results suggest that complex disease states do not significantly contribute to diagnostic misclassification, but may actually enhance the ability to characterise lesions.

Despite the increased challenge in scanning these patients and mapping all disease sites, we suspect the unexpected reduction in sensitivity observed upon exclusion is a product of the enhanced critical investigative nature of the sonologist, instigated by the presence of obliteration and/or DE of the bowel. In other words, a *red flag* is raised when these severe states are recognised, and the sonologist makes concerted efforts to map other adjacent pathology. Moreover, the maintenance of strong diagnostic performance may also be secondary to the posterior TVUS approach technique with the positioning of the probe in the posterior vaginal fornix, which allows for closer proximity to disease sites. However, the observed reduction in sensitivity, decreasing by as much as 6.4%, suggests that the absence of complex disease states may hinder lesion detection. This implies that their presence may enhance, rather than impair, sonographic recognition of pathology. These findings underscore the need for a systematic and targeted approach to TVUS, emphasising integrating highly reproducible techniques into routine assessments [9]. Methods such as the sliding sign between the posterior uterus and bowel should be consistently applied to enhance diagnostic reliability and improve lesion detection, particularly in cases where deep endometriosis is suspected [2]. Standardising these approaches in everyday practice may mitigate variability in operator performance and optimise the overall accuracy of TVUS in characterising disease extent.

Reflecting on deficiencies in diagnostic test accuracy, we may consider how the anatomical environment and landmarks are heavily distorted in severe endometriosis, potentially yielding a discrepancy in where a nodule is labelled at ultrasound versus surgery. Normalising anatomy through adhesiolysis and excision of other pathology (e.g., large endometriomas) throughout the surgery may result in a more accurate description of the location of the disease. However, it does not mean the ultrasound test did not correctly identify the presence of endometriosis; instead, it only incorrectly labelled the location. Regardless of training or expertise, it is most likely that this type of confusion will exist with the retrocervical structures, the USLs and TU and potentially the peritoneum of the POD, which is in continuity with these structures. A potential solution to this dilemma is considering the entire USL/TU complex as one retrocervical complex (or a ‘horseshoe region’). This may be particularly important in settings where the surgeon is not the sonologist, and discrepancies in localisation of a deposit could hypothetically result in false negative classifications of disease locations at surgery or unnecessary excision of regions believed to be impacted by DE. Similarly, the bowel and vagina have distinctive anatomical landmarks, even in the context of severe disease, so it is our belief that these regions should be distinctly assessed and characterised, producing more clarity on disease localisation.

### 4.3 Strengths and Limitations

This study benefits from adherence to the STARD guidelines, ensuring completeness and transparency of reported diagnostic accuracies. Additionally, prospectively recruited participants and using high‐quality ultrasound and surgical equipment benefit the study. The study also benefits from the novel exploration of the impact of complex disease states on the diagnostic accuracy of TVUS diagnosis of DE of the USLs and TU.

Despite these strengths, several limitations must be acknowledged. Generalisability may be affected, as a single highly trained surgeon‐sonologist performed both TVUS and the corresponding surgeries. While using a single expert surgeon‐sonologist minimised inter‐operator variability, it may have introduced diagnostic review bias, as the operator was not blinded during surgery. This design also introduces potential recall and confirmation biases, whereby prior knowledge of ultrasound findings may influence intraoperative interpretation, or vice versa. Future studies should incorporate blinded assessments and involve operators with varying levels of expertise to improve external validity and real‐world applicability. Nevertheless, a recent study by Maple et al. [13] demonstrated high inter‐ and intra‐observer agreement among sonographers and radiologists in general imaging departments when reviewing stored ultrasound images of the USLs, suggesting that once the USLs are correctly identified, interpretation of their features may be reproducible. However, it is essential to note that the study was based on off‐line image review, not real‐time scanning. In practice, the initial identification of the USLs remains a significant technical challenge for many operators and may contribute substantially to variability in diagnostic performance. Furthermore, the prevalence of endometriosis in our study population, representing a tertiary referral surgical cohort, was high, with many patients having prior endometriosis surgery or severe disease manifestations such as POD obliteration. This may have contributed to the elevated PPVs observed, particularly for USL and TU involvement. As the PPVs are dependent on disease prevalence, these values may not generalise to outpatient radiology settings or general gynaecology clinics where disease prevalence is lower.

## 5 Conclusion

The USLs and TU are the most common locations for DE and often co‐exist with complex disease states, including POD obliteration and DE of the bowel. Our findings highlight that the presence of complex disease states does not reduce overall diagnostic accuracy but instead may enhance sensitivity by prompting a more meticulous sonographic assessment. The observed decrease in sensitivity upon excluding these cases suggests that lesion detectability may be aided by the structural and investigative cues provided by severe disease. These results emphasise the importance of recognising complex pathology as a diagnostic prompt rather than an obstacle, reinforcing the need for a systematic and targeted approach in TVUS assessments of DE.

## Author Contributions

S.M.F. and M.L. contributed equally to this work. Both authors were involved in the conception, study design, data collection, analysis and manuscript drafting. Both authors have reviewed and approved the final version of the manuscript and agree to be accountable for all aspects of the work.

## Disclosure

Shay Freger reports advocacy work with TENC and EndoACT and grants with CIHR and CanSAGE outside submitted work. Mathew Leonardi reports grants from Australian MRFF, AbbVie, CanSAGE, CIHR, Hamilton Health Sciences, Hyivy, Pfizer; honoraria for lectures/writing from AIUM, GE Healthcare, Bayer, AbbVie, consultancy work with AbbVie, Hologic, Chugai, Gesynta, Roche Diagnostics, Afynia, Pfizer, affiliations with Imagendo, SUGO—Specialised Ultrasound in Gynaecology & Obstetrics, outside the submitted work.

## Conflicts of Interest

The authors declare no conflicts of interest.

## Supporting information


**Video S1:** Sonographic navigation of DE of the USLs and TU in the presence of POD using dynamic pressure.

## Data Availability

The data that support the findings of this study are available from the corresponding author upon reasonable request.
